# Trends in Disease Burden Attributable to Tobacco in China, 1990–2017: Findings From the Global Burden of Disease Study 2017

**DOI:** 10.3389/fpubh.2020.00237

**Published:** 2020-07-09

**Authors:** Haoyu Wen, Cong Xie, Fang Wang, Yini Wu, Chuanhua Yu

**Affiliations:** ^1^Department of Preventive Medicine, School of Health Sciences, Wuhan University, Wuhan, China; ^2^Hubei Center for Disease Control and Prevention, Wuhan, China; ^3^Department of Nursing, School of Health Sciences, Wuhan University, Wuhan, China; ^4^China Global Health Institute, Wuhan University, Wuhan, China

**Keywords:** tobacco, global burden of disease, deaths, disability-adjusted life years, smoking, secondhand smoke, chewing tobacco

## Abstract

In 2018, there were more than 371 million cigarette smokers and 12. 6 million electronic cigarette users, with 340.2 million non-smokers exposed to secondhand smoke (SHS) in China, which resulted in heavy tobacco-attributable disease burden. According to the definition by the Global Burden of Disease Study 2017 (GBD 2017), tobacco is a level 2 risk factor that consists of three sublevel risk factors, namely, smoking, SHS, and chewing tobacco. In this study, we aimed to evaluate the trends in deaths and disability-adjusted life years (DALYs) attributable to tobacco, smoking, SHS, and chewing tobacco by sex in China from 1990 to 2017 and to explore the leading causes of tobacco-attributable deaths and DALYs using data from the GBD 2017. From 1990 to 2017, the tobacco-attributable death rates per 100,000 people decreased from 75.65 [95% uncertainty interval (95% UI) = 56.23–97.74] to 70.90 (95% UI = 59.67–83.72) in females and increased from 198.83 (95% UI = 181.39–217.47) to 292.39 (95% UI = 271.28–313.76) in males. From 1990 to 2017, the tobacco-attributable DALY rates decreased from 2209.11 (95% UI = 1678.63–2791.91) to 1489.05 (95% UI = 1237.65–1752.57) in females and increased from 5650.42 (95% UI = 5070.06–6264.39) to 6994.02 (95% UI = 6489.84–7558.41) in males. In 2017, the tobacco-attributable deaths in China were concentrated on chronic obstructive pulmonary disease, ischemic heart disease, lung cancer, and stroke. The focus of tobacco control for females was SHS in 1990, whereas smoking and SHS were equally important for tobacco control in females in 2017. Increasing tobacco taxes and prices may be the most effective and feasible measure to reduce tobacco-attributable disease burdens.

## Introduction

According to the definition by the Global Burden of Disease Study 2017 (GBD 2017), tobacco is a level 2 risk factor that consists of three sublevel risk factors, namely, smoking, secondhand smoke (SHS), and chewing tobacco (1). Tobacco has long served as the largest preventable cause of death worldwide (2–4). China has been the largest tobacco producer worldwide, with only 1% of cigarettes produced in China being exported and the rest being consumed domestically (5). More than 371 million cigarette smokers and 12.6 million electronic cigarette (e-cigarette) users contributed to the high tobacco consumption in China; furthermore, 340.2 million non-smokers were exposed to SHS in China in 2018 (6). In 2017, 60.73 million disability-adjusted life years (DALYs) were recorded, and 2.60 million deaths could be attributable to tobacco in China (7). Tobacco is a crucial risk factor for the leading causes of death in China, such as chronic obstructive pulmonary disease (COPD), lung cancer, stroke, and ischemic heart disease (IHD) (8–11). Given the large number of populations affected by tobacco and the heavy tobacco-attributable disease burdens in China, a comprehensive study of the tobacco-attributable disease burden is urgently needed.

Most of the current studies on tobacco in China have focused on smoking prevalence and SHS exposure and the death attributable to them (12–15). However, few studies have paid attention to chewing tobacco. Furthermore, previous studies did not include the latest data. In addition, none of the studies synthesized the disease burden attributable to smoking, SHS, and chewing tobacco in China and explored the root cause for the trends in the tobacco-attributable disease burdens from 1990 to 2017.

To address these limitations, the present work aimed to evaluate the trends in deaths and DALYs attributable to tobacco, smoking, SHS, and chewing tobacco by sex in China from 1990 to 2017. Furthermore, we explored the leading causes of deaths and DALYs attributable to tobacco in 1990 and 2017, which could improve the focus of our tobacco control program.

## Materials and Methods

### Data Sources

The data for this study were obtained from the GBD 2017, which estimated the levels and trends in exposure, attributable deaths, and attributable DALYs by sex and year for 84 behavioral, environmental and occupational, and metabolic risks or groups of risks between 1990 and 2017 (1). In the present work, we measured the tobacco-attributable disease burdens by deaths and DALYs, which was a comprehensive indicator to measure the disease burden. Years of life lost due to premature death (YLLs), an indicator that measured the burden of premature death, was calculated as the sum of the standard life expectancy of each death at the age of death. Years lived with disability (YLDs), an indicator that quantified the burden of non-fatal health outcomes, was estimated as the product of a disability weight for the health states of each sequela and prevalence and then adjusted for comorbidity. DALYs was the sum of YLLs and YLDs. In the GBD 2017, the health outcome data were reported based on the World Health Organization clinical criteria and the 9th and 10th revisions of the International Classification of Disease. The tobacco-attributable age-standardized death rates (ASDRs) and the DALY rates were based on the GBD 2017 global age-standard population.

GBD 2017 requires original data on the fatal and non-fatal outcomes in China to measure the tobacco-attributable disease burdens in the country. For the fatal outcomes in China, the original data were gathered primarily from five data sources (16): censuses, surveys (including the Annual Survey on Population Change and the Intercensal Survey), surveillance systems (including the Disease Surveillance Point System and the Maternal and Child Surveillance System), the Chinese Center for Disease Control and Prevention Cause of Death Reporting System, and the China Cancer Registry. For the non-fatal outcomes in China, the original data were obtained from published studies, hospital inpatient data, and the aforementioned five data sources. The GBD cause of death ensemble modeling tool and cause of death correction procedure and DisMod-MR 2.1 were used to make consistent and accurate estimates.

### Estimating Tobacco-Attributable Disease Burden

Through the above data sources and methods, we can estimate the DALYs and death for health outcomes in China. GBD 2017 estimated the tobacco-attributable DALYs as the total DALYs multiplied by the population-attributable fraction (PAF) for the tobacco-related risk–outcome pair by age, sex, and year. The method for estimating the tobacco-attributable deaths is similar to those of the DALYs. The PAF for each individual risk–outcome pair was estimated independently. The estimates of PAF are based on Chinese tobacco exposure data.

The original data of tobacco exposure were obtained from diversified sources. These sources are cross-sectional nationally representative household surveys; household composition module, including the Demographic Health Surveys, Multiple Indicator Cluster Surveys, and Living Standards Measurement Surveys, self-reported tobacco exposure from cross-sectional surveys, including the Global Adult Tobacco Surveys and WHO STEPS Surveys; national and subnational censuses; and published studies. Spatiotemporal Gaussian process regression was used to estimate PAF.

Two points must be declared. Firstly, in the estimate of the smoking-attributable disease burden, we used the 5-year-lagged smoking prevalence as the exposure. Secondly, for GBD 2017, the current definition of chewing tobacco is the definition of smokeless tobacco in the previous version of the GBD study, including snuff and chewing tobacco and e-cigarettes. Detailed methods for the estimation of the tobacco-attributable burden are presented in published studies (4).

### Uncertainty Analysis

We captured and propagated uncertainty through all steps of the analysis, including sampling uncertainty from data extraction, uncertainty in the ST-GPR model, and uncertainty in deaths and DALYs for the 38 tobacco-related health outcomes. All estimated numbers and rates of the tobacco-attributable deaths and DALYs in this study were reported with the 95% uncertainty interval (UI). The 95% UI was estimated by *a posteriori* simulation of 1,000 samples, whose upper and lower bounds were derived based on the 2.5th and 97.5th percentiles of the uncertainty distribution.

## Results

### Tobacco-Attributable Deaths

Table 1 presents the values and percentage changes of the tobacco-attributable deaths and DALYs from 1990 to 2017. The number of tobacco-attributable deaths was considerably more in males than in females between 1990 and 2017. In 1990, 438.38 (95% UI = 325.87–566.41) thousand deaths in females and 1227.74 (95% UI = 1120.06–1342.83) thousand deaths in males could be attributed to tobacco. From 1990 to 2017, the tobacco-attributable deaths increased by 11.80 and 71.76% in females and males, respectively, which led to an increasing sex gap for tobacco-attributable deaths. In 2017, 490.12 (95% UI = 412.49–578.73) and 2108.73 (95% UI = 1956.45–2262.82) thousand tobacco-attributable deaths occurred in females and males, respectively. Notably, the number of smoking-attributable deaths exceeded that of SHS in females in 2017.

**Table 1:** Values and percentage changes of the tobacco-attributable deaths and DALYs from 1990 to 2017.

	**Females**			**Males**		
	**1990 (95%CI)**	**2017 (95%CI)**	**1990–2017**	**1990 (95%CI)**	**2017 (95%CI)**	**1990–2017**
			**%change**			**%change**
**All age deaths (in thousands)**						
Tobacco	438.38 (325.87–566.41)	490.12 (412.49–578.73)	11.8	1227.74 (1120.06–1342.83)	2108.73 (1956.45–2262.82)	71.76
Smoking	177.58 (129.46–234.27)	264.72 (236.07–297.39)	49.07	1066.86 (999.14–1135.38)	1932.93 (1825.18–2036.58)	81.18
SHS	254.27 (192.67–321.85)	218.35 (172.34–270.35)	−14.13	155.75 (118.20–199.34)	168.30 (126.99–214.57)	8.06
Chewing tobacco	6.53 (3.74–10.28)	7.05 (4.08–10.99)	8.02	5.14 (2.72–8.11)	7.50 (4.28–11.68)	46.09
**All age death rate per 100,000**						
Tobacco	75.65 (56.23–97.74)	70.90 (59.67–83.72)	−6.28	198.83 (181.39–217.47)	292.39 (271.28–313.76)	47.06
Smoking	30.64 (22.34–40.43)	38.29 (34.15–43.02)	24.96	172.77 (161.81–183.87)	268.02 (253.08–282.39)	55.13
SHS	43.88 (33.25–55.54)	31.59 (24.93–39.11)	−28.02	25.22 (19.14–32.28)	23.34 (17.61–29.75)	−7.48
Chewing tobacco	1.13 (0.65–1.77)	1.02 (0.59–1.59)	−9.45	0.83 (0.44–1.31)	1.04 (0.59–1.62)	25.08
**Age-standardized death rate per 100,000**						
Tobacco	116.57 (85.45–152.06)	53.75 (45.27–63.61)	−53.89	355.43 (321.88–391.29)	246.26 (228.15–265.54)	−30.72
Smoking	49.93 (36.22–66.90)	29.11 (25.97–32.85)	−41.70	310.43 (289.88–332.20)	224.25 (211.67–236.88)	−27.76
SHS	65.04 (48.29–82.53)	23.91 (18.88–29.60)	−63.24	43.73 (31.32–57.02)	21.21 (16.03–27.39)	−51.5
Chewing tobacco	1.61 (0.93–2.63)	0.73 (0.42–1.15)	−54.33	1.28 (0.68–2.07)	0.80 (0.45–1.27)	−37.41
**All age DALYs (in thousands)**						
Tobacco	12801.62 (9727.53–16178.89)	10293.64 (8555.77–12115.36)	−19.59	34890.71 (31307.07–38681.91)	50440.27 (46804.15–54510.59)	44.57
Smoking	3520.02 (2545.57–4557.75)	4851.43 (4287.89–5415.64)	37.82	28085.81 (26165.06–29985.51)	46335.29 (43683.04–49272.93)	64.98
SHS	9143.77 (7102.97–11407.10)	5311.40 (4189.69–6502.21)	−41.91	6675.30 (5074.29–8491.56)	3932.81 (3024.39–4973.93)	−41.08
Chewing tobacco	137.83 (79.00–214.04)	130.81 (78.19–197.51)	−5.09	129.60 (67.72–204.85)	172.17 (96.72–263.74)	32.85
**All age DALY rate per 100,000**						
Tobacco	2209.11 (1678.63–2791.91)	1489.05 (1237.65–1752.57)	−32.60	5650.42 (5070.06–6264.39)	6994.02 (6489.84–7558.41)	23.78
Smoking	607.43 (439.28–786.51)	701.79 (620.27–783.41)	15.53	4548.39 (4237.33–4856.04)	6424.83 (6057.07–6832.16)	41.25
SHS	1577.90 (1225.73–1968.47)	768.33 (606.07–940.59)	−51.31	1081.04 (821.76–1375.18)	545.32 (419.36–689.68)	−49.56
Chewing tobacco	23.78 (13.63–36.94)	18.92 (11.31–28.57)	−20.44	20.99 (10.97–33.17)	23.87 (13.41–36.57)	13.74
**Age-standardized DALY rate per 100,000**						
Tobacco	2749.54 (2098.47–3482.49)	1081.26 (899.62–1271.70)	−60.67	7843.74 (7106.97–8636.20)	5261.67 (4878.51–5684.44)	−32.92
Smoking	851.15 (631.28–1101.39)	500.89 (442.94–558.67)	−41.15	6525.72 (6100.38–6954.52)	4794.50 (4518.07–5090.26)	−26.53
SHS	1867.44 (1449.27–2332.85)	567.42 (448.92–693.57)	−69.61	1289.62 (991.70–1636.57)	450.17 (350.74–568.05)	−65.09
Chewing tobacco	30.94 (17.92–48.24)	12.95 (7.76–19.46)	−58.14	28.40 (14.89–45.11)	17.01 (9.70–26.13)	−40.11
**All age YLL rate per 100,000**						
Tobacco	1949.17 (1,485–2461.45)	1149.05 (964.59–1353.02)	−41.05	5050.44 (4538.06–5619.18)	6039.25 (5590.59–6483.59)	19.58
Smoking	521.45 (378.49–676.17)	562.47 (499.33–630.37)	7.86	4006.61 (3753.96–4282.25)	5548.17 (5221.34–5854.46)	38.48
SHS	1404.25 (1093.07–1748.83)	568.14 (454.22–694.75)	−59.54	1023.07 (773.26–1304.1)	467.58 (356.06–593.1)	−54.3
Chewing tobacco	23.46 (13.44–36.45)	18.45 (11.03–27.9)	−21.38	20.76 (10.84–32.83)	23.5 (13.19–36.04)	13.19
**Age-standardized YLL rate per 100,000**						
Tobacco	2423.67 (1844.94–3065.39)	838.56 (704.40–986.60)	−65.4	7045.07 (6397.00–7770.21)	4544.81 (4206.11–4879.52)	−35.49
Smoking	734.55 (541.05–950.69)	401.83 (356.86–450.39)	−45.3	5803.81 (5447.01–6185.77)	4138.47 (3899.27–4362.15)	−28.69
SHS	1658.60 (1286.28–2067.12)	424.11 (339.98–517.25)	−74.43	1213.18 (935.29–1539.82)	389.61 (297.29–491.67)	−67.89
Chewing tobacco	30.51 (17.61–47.58)	12.62 (7.57–18.97)	−58.63	28.08 (14.71–44.63)	16.73 (9.55–25.69)	−40.41
**All age YLD rate per 100,000**						
Tobacco	259.95 (171.84–353.66)	340 (237.8–443.84)	30.8	599.98 (444.08–759.42)	954.77 (711.81–1212.98)	59.13
Smoking	85.98 (58.07–119.99)	139.33 (107.54–170.86)	62.05	541.78 (408.37–676.23)	876.66 (664.7–1096.39)	61.81
SHS	173.65 (113.59–233.16)	200.19 (129.98–272.22)	15.29	57.97 (35.59–82.82)	77.74 (46.92–115.99)	34.11
Chewing tobacco	0.32 (0.18–0.51)	0.48 (0.28–0.76)	48.39	0.22 (0.11–0.38)	0.37 (0.2–0.6)	65.46
**Age-standardized YLD rate per 100,000**						
Tobacco	325.87 (216.46–440.80)	242.70 (170.07–316.52)	−25.52	798.66 (598.22–1001.47)	716.86 (535.81–908.36)	−10.24
Smoking	116.60 (80.43–160.36)	99.06 (76.68–121.44)	−15.04	721.91 (551.63–892.07)	656.03 (498.90–819.20)	−9.13
SHS	208.84 (135.78–279.75)	143.32 (93.19–194.55)	−31.38	76.43 (46.43–108.86)	60.56 (36.76–88.71)	−20.76
Chewing tobacco	0.43 (0.25–0.69)	0.33 (0.20–0.53)	−23.66	0.32 (0.16–0.54)	0.27 (0.15–0.45)	−14.38

*The unit for DALYs, YLLs, and YLDs is person-years*.

*95% CI, 95% confidence interval; YLL, year of life lost; YLD, year lived with disability; DALY, disability-adjusted life year; SHS, secondhand smoke*.

**Figure 1:**
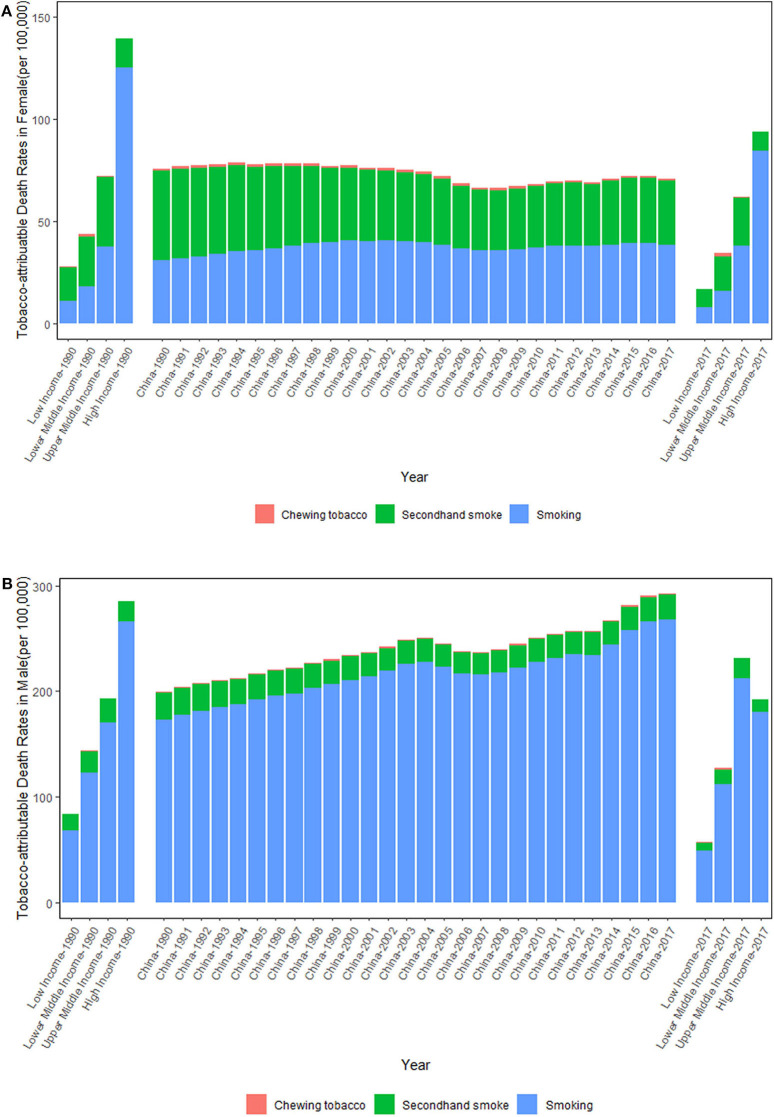
Trends in the tobacco-attributable death rates per 100,000 people in Chinese females **(A)** and males **(B)** from 1990 to 2017. Income-divided countries from the World Bank.

Figure 1 shows the trends of the tobacco-attributable death rates per 100,000 people from 1990 to 2017. From 1990 to 2017, the tobacco-attributable death rates increased significantly in males and decreased slightly in females. For females, the tobacco-attributable death rates decreased by 6.28%, from 75.65 (95% UI = 56.23–97.74) in 1990 to 70.90 (95% UI = 59.67–83.72) in 2017. For males, the tobacco-attributable death rates increased by 47.06%, from 198.83 (95% UI = 181.39–217.47) in 1990 to 292.39 (95% UI = 271.28–313.76) in 2017. In comparison with World Bank income-divided countries, the tobacco-attributable death rates in China were close to those of upper-middle-income countries in 1990. In 2017, the tobacco-attributable death rates in Chinese females were close to those of upper-middle-income countries, but the rates in Chinese males were significantly higher than those of upper-middle-income countries. It is noteworthy that the smoking-attributable death rates increased in both sexes, whereas the SHS-attributable death rates decreased in both sexes from 1990 to 2017. From 1990 to 2017, the smoking-attributable death rates increased by 24.96 and 55.13% in females and males, respectively, and the SHS-attributable death rates decreased by 28.02 and 7.48% in females and males, respectively. The chewing tobacco-attributable death rates varied within a narrow range in both sexes between 1990 and 2017 overall.

**Figure 2:**
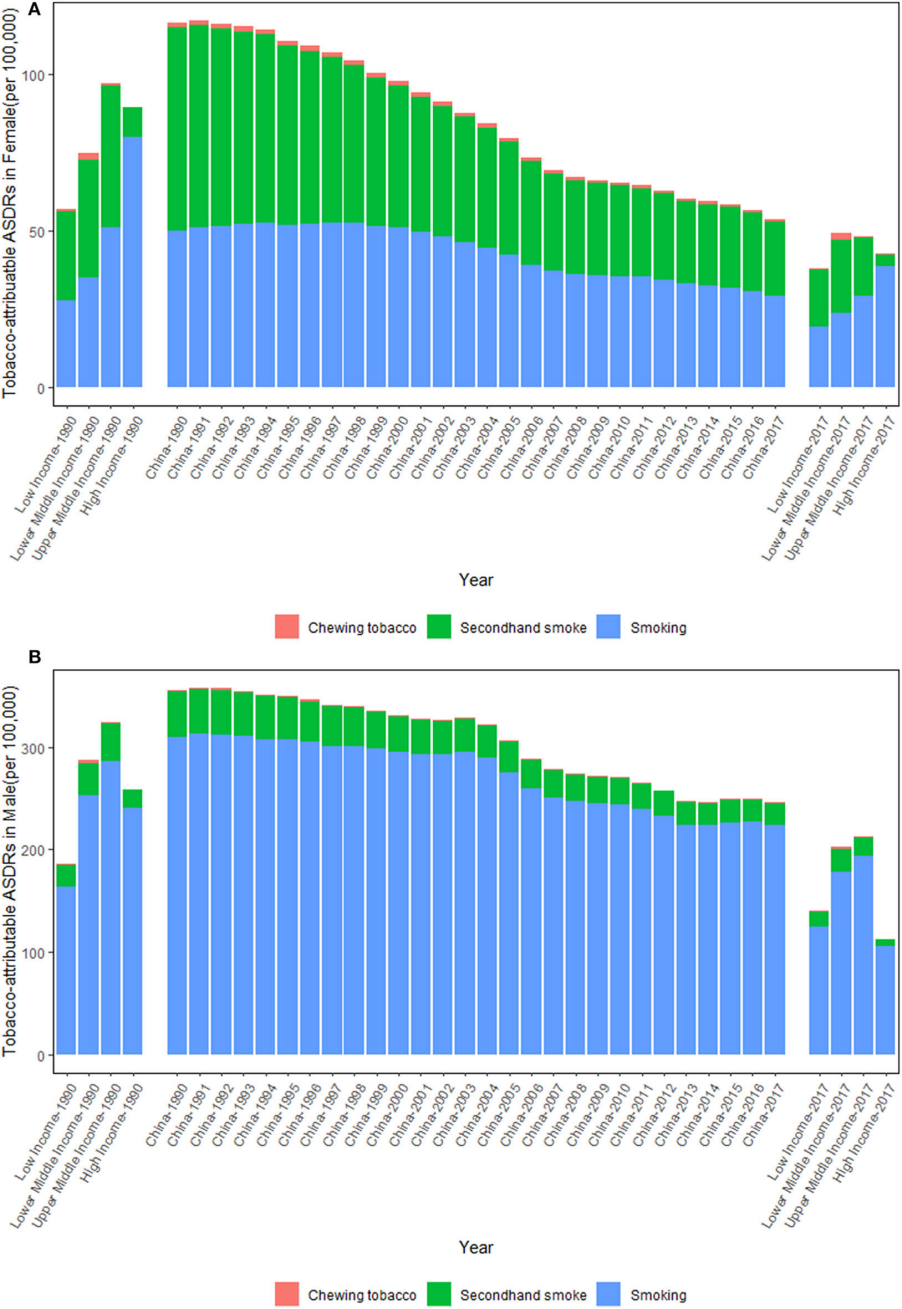
Trends in the tobacco-attributable age-standardized death rates (ASDRs) per 100,000 people in Chinese females **(A)** and males **(B)** from 1990 to 2017. Income-divided countries from the World Bank.

Figure 2 shows the trends in the tobacco-attributable ASDRs per 100,000 people from 1990 to 2017. In comparison to the increasing tobacco-attributable deaths between 1990 and 2017, the tobacco-attributable ASDRs showed downward trends in both sexes, but were more significant in females. Although the tobacco-attributable ASDRs in China decreased continuously from 1990 to 2017 in both sexes, they were still higher compared with those of all World Bank income-divided countries. For females, the tobacco-attributable ASDRs per 100,000 people decreased by 53.89%, from 116.57 (95% UI = 85.45–152.06) in 1990 to 53.75 (95% UI = 45.27–63.61) in 2017, whereas for males, the rates decreased by 30.72%, from 355.43 (95% UI = 321.88–391.29) in 1990 to 246.26 (95% UI = 228.15–265.54) in 2017.

### Tobacco-Attributable DALYs

As shown in Table 1, the tobacco-attributable DALYs were consistently higher in males than in females. From 1990 to 2017, the tobacco-attributable DALYs decreased by 11.97% in females and increased by 44.54% in males, which led to an increasing sex gap for the tobacco-attributable DALYs. In 2017, the tobacco-attributable DALYs were 10293.64 (95% UI = 8555.77–12115.36) thousand person-years in females, which decreased by 19.59% from 12801.62 (95% UI = 9727.53–16178.89) thousand person-years in 1990. For males, the tobacco-attributable DALYs increased by 44.57%, from 34890.71 (95% UI = 31307.07–38681.91) thousand person-years in 1990 to 50440.27 (95%UI = 46804.15–54510.59) thousand person-years in 2017. It should be pointed out that the number of smoking-attributable DALYs were close to those of SHS in females in 2017. Although the number of YLDs were smaller than those of YLLs, these are becoming increasingly important. In 1990, the YLL/YLD ratio in females was 7.44 and was 8.82 in males, which decreased to 3.46 and 6.34 in 2017, respectively.

**Figure 3:**
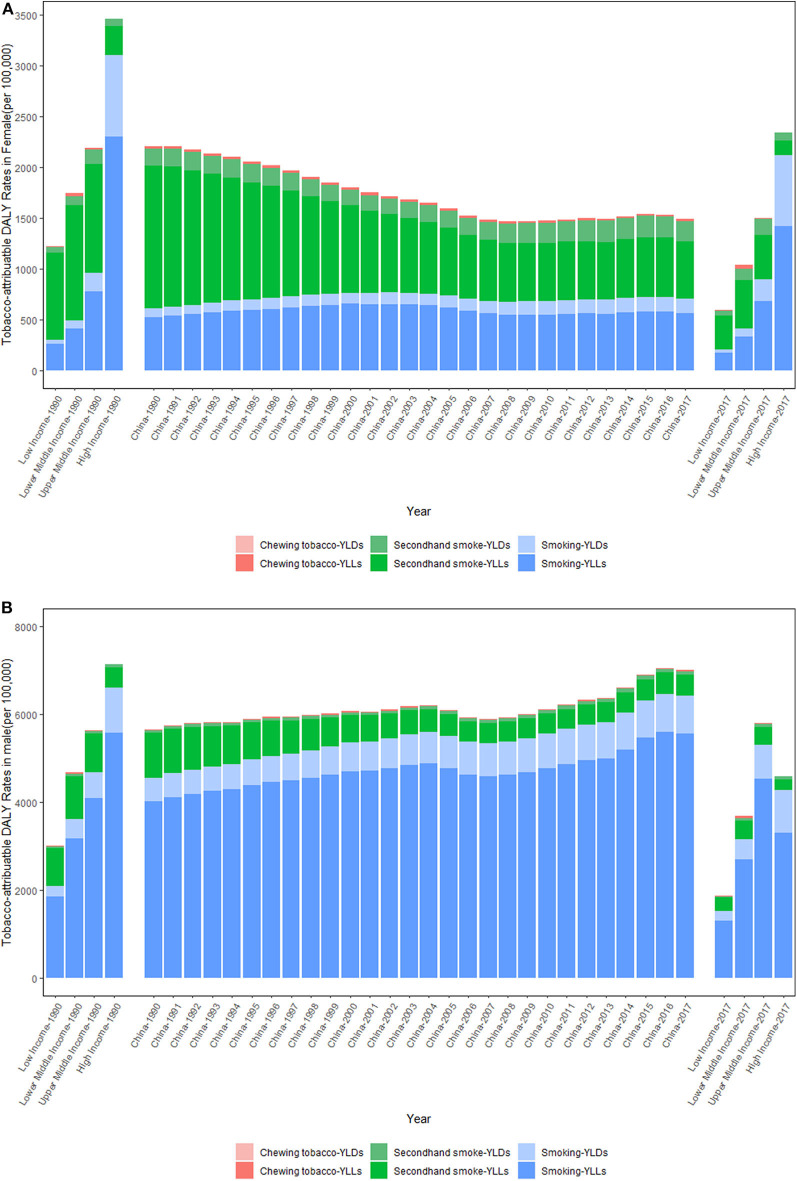
Trends in the tobacco-attributable DALY rates per 100,000 people in Chinese females **(A)** and males **(B)** from 1990 to 2017. *DALY*, disability-adjusted life year; *YLL*, years of life lost due to premature death; *YLD*, years lived with disability. Income-divided countries from the World Bank.

Figure 3 shows the trends of the tobacco-attributable DALY rates per 100,000 people from 1990 to 2017. The trends of the tobacco-attributable DALY rates were similar to those of the tobacco-attributable death rates. That is, the tobacco-attributable DALY rates increased in males and decreased in females. For females, the tobacco-attributable DALY rates decreased by 32.60%, from 2209.11 (95% UI = 1678.63–2791.91) in 1990 to 1489.05 (95% UI = 1237.65–1752.57) in 2017. For males, the tobacco-attributable DALY rates increased by 23.78%, from 5650.42 (95% UI = 5070.06–6264.39) in 1990 to 6994.02 (95% UI = 6489.84–7558.41) in 2017. The comparison of World Bank income-divided countries and China for tobacco-attributable DALY rates was similar for tobacco-attributable death rates. The tobacco-attributable DALY rates in China were close to those of upper middle-income countries in 1990. In 2017, the tobacco-attributable DALY rates in Chinese females were close to those of upper middle-income countries, but the rates in Chinese males were significantly higher than those of upper middle-income countries. For the three level 3 tobacco-related risks, the smoking-attributable DALY rates increased in both sexes, whereas the SHS-attributable DALY rates decreased in both sexes between 1990 and 2017. From 1990 to 2017, the smoking-attributable DALY rates increased by 15.53 and 41.25% in females and males, respectively, and the SHS-attributable DALY rates decreased by 51.31 and 49.56% in females and males, respectively. The chewing tobacco-attributable DALY rates decreased in females and increased in males. Notably, the YLD rates attributable to tobacco, smoking, SHS, and chewing tobacco all increased in both sexes from 1990 to 2017.

**Figure 4:**
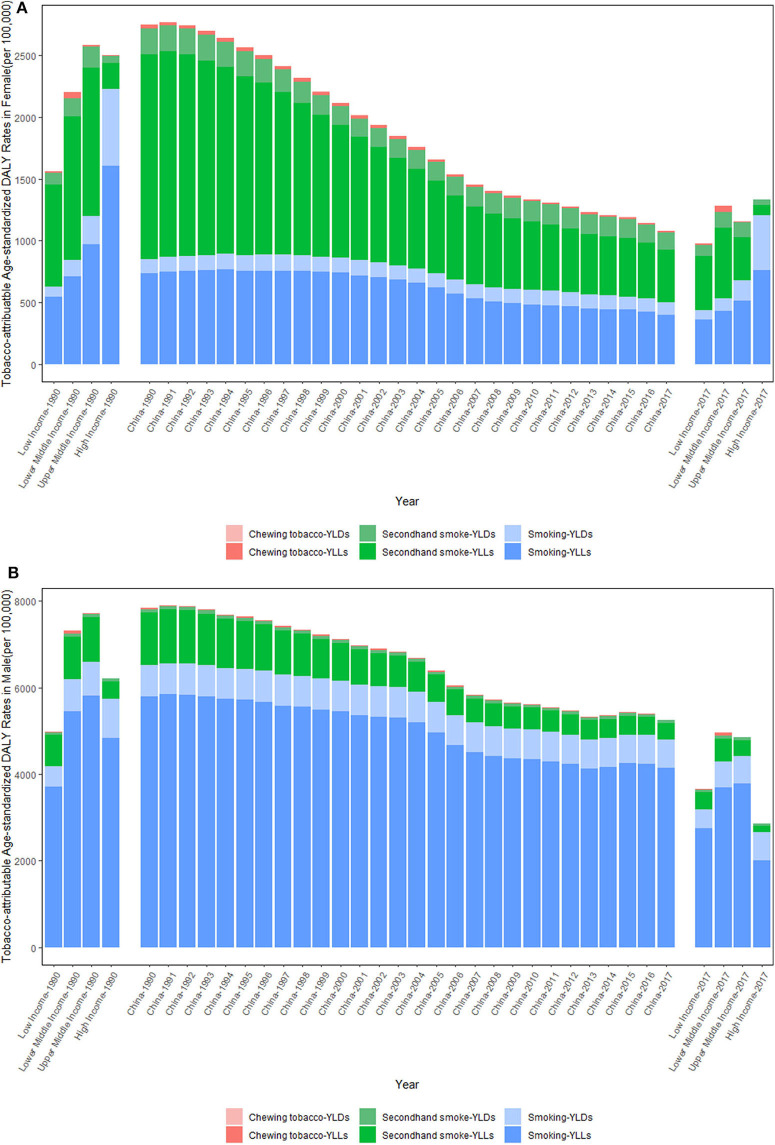
Trends in the tobacco-attributable age-standardized DALY rates per 100,000 people in Chinese females **(A)** and males **(B)** from 1990 to 2017. *DALY*, disability-adjusted life year; *YLL*, years of life lost due to premature death; *YLD*, years lived with disability. Income-divided countries from the World Bank.

Figure 4 shows the trends in the tobacco-attributable age-standardized DALY rates per 100,000 people from 1990 to 2017. Between 1990 and 2017, the tobacco-attributable age-standardized DALY rates showed downward trends in both sexes, but were more significant in females. For females, the tobacco-attributable age-standardized DALY rates decreased by 60.67%, from 2749.54 (95% UI = 2098.47–3482.49) in 1990 to 1081.26 (95% UI = 899.62–1271.70) in 2017, whereas for males, the rates decreased by 32.92%, from 7843.74 (95% UI = 7106.97–8636.20) in 1990 to 5261.67 (95% UI = 4878.51–5684.44) in 2017.

### Causes for Tobacco-Attributable Disease Burdens

**Figure 5:**
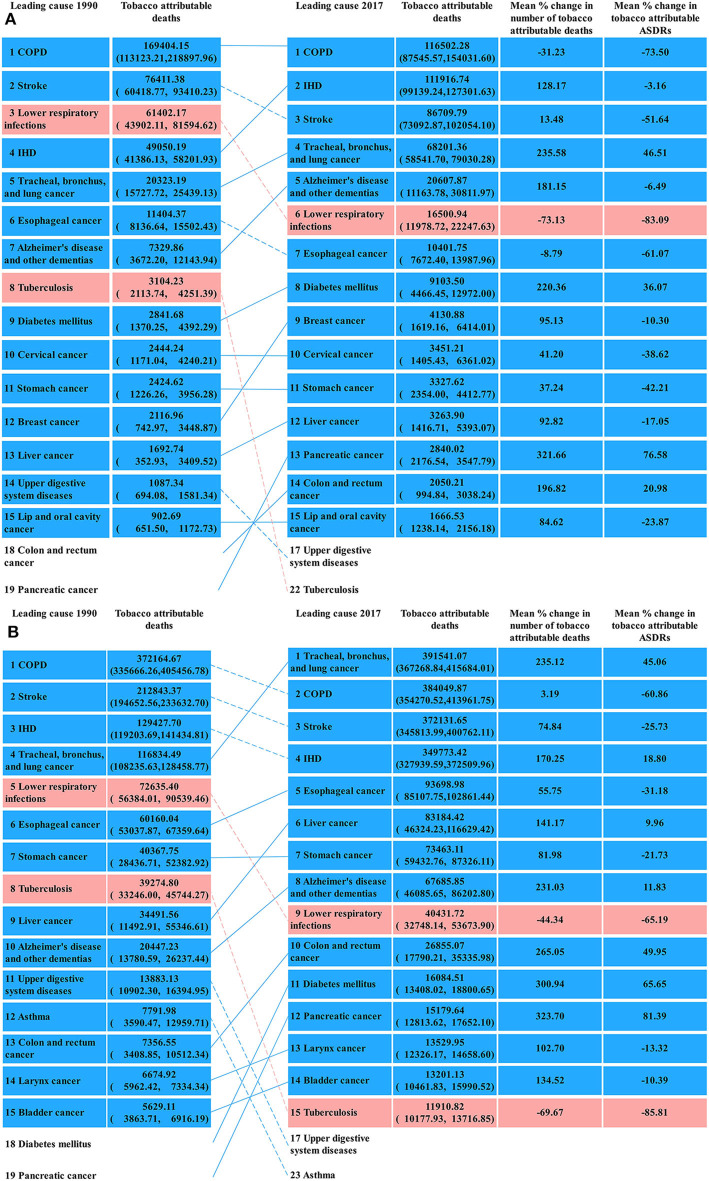
Top 15 causes of tobacco-attributable deaths in Chinese females **(A)** and males **(B)** in 1990 and 2017.

The cause for the tobacco-attributable deaths was similar to that for the tobacco-attributable DALYs. Thus, we only showed the results of the former in the see section Results and presented the latter as Supplementary Material. Figure 5 presents the top 15 causes of the tobacco-attributable deaths in 1990 and 2017. For females, COPD (169.40 thousand, 95% UI = 113.12–218.90), stroke (76.41 thousand, 95% UI = 60.42–93.41), lower respiratory infections (61.40 thousand, 95% UI = 43.90–81.59), and IHD (49.05 thousand, 95% UI = 41.39–58.20) were the predominant causes of tobacco-attributable deaths in 1990, accounting for 85.36% of the tobacco-attributable deaths. In 2017, the predominant causes of tobacco-attributable deaths in females were COPD (116.50 thousand, 95% UI = 87.55–154.03), IHD (111.92 thousand, 95% UI = 99.14–127.30), stroke (86.71 thousand, 95% UI = 73.09–102.05), and tracheal, bronchus, and lung cancer (68.20 thousand, 95% UI = 58.54–79.03), which accounted for 82.03% of the tobacco-attributable deaths in females in 2017. Lower respiratory infection was a leading cause of tobacco-attributable deaths in females in 1990, whereas tracheal, bronchus, and lung cancer replaced it as a leading cause in 2017.

For males, COPD (372.16 thousand, 95% UI = 335.67–405.46), stroke (212.84 thousand, 95% UI = 194.65–233.63), IHD (129.43 thousand, 95% UI = 119.20–141.43), and tracheal, bronchus, and lung cancer (116.83 thousand, 95% UI = 108.24–128.46) were the predominant causes of tobacco-attributable deaths in 1990, accounting for 71.11% of the tobacco-attributable deaths. In 2017, the predominant causes of tobacco-attributable deaths in males were tracheal, bronchus, and lung cancer (391.54 thousand, 95% UI = 367.27–415.68); COPD (384.05 thousand, 95% UI = 354.27–413.96), stroke (372.13 thousand, 95% UI = 345.81–400.76), and IHD (349.77 thousand, 95% UI = 327.94–372.51), accounting for 74.18% of the tobacco-attributable deaths in males. For both sexes, lower respiratory infection and tuberculosis held the largest percentage decline in the top 15 causes of tobacco-attributable deaths from 1990 to 2017. After synthesizing tobacco-attributable deaths in both sexes in 2017, we found that the tobacco-attributable deaths were concentrated on COPD, IHD, tracheal, bronchus, and lung cancer, and stroke, whose deaths accounted for 75.66% of the total tobacco-attributable deaths in both sexes.

Table 2 shows the proportion of tobacco-attributable deaths to the total deaths for the top 15 causes of death in 1990 and 2017. The proportion was considerably higher in males than in females. For males, tobacco-attributable death accounted for an astonishingly high proportion of death from leading causes (30.88, 37.07, 69.34, and 82.04% for stroke, IHD, COPD, and lung cancer, respectively). For females, the proportion was relatively low, with COPD, tracheal, bronchus, and lung cancer, and lower respiratory infections being the only three causes whose proportion was more than 20% in 2017. The proportion varied within a narrow range in both sexes between 1990 and 2017 overall.

**Table 2:** Proportion of tobacco-attributable deaths to the total deaths for the top 10 death causes in 1990 and 2017.

**Proportion of tobacco-attributable deaths to the total deaths in 1990**			**Proportion of tobacco-attributable deaths to the total deaths in 2017**		
**Top 15 death causes in 1990**	**Females (%)**	**Males (%)**	**Top 15 death causes in 2017**	**Females (%)**	**Males (%)**
1. Stroke	12.32	32.86	1. Stroke	9.58	30.88
2. COPD	28.69	62.95	2. IHD	13.88	37.07
3. IHD	18.29	41.43	3. COPD	28.28	69.34
4. Lower respiratory infections	23.13	27.18	4. Tracheal, bronchus, and lung cancer	31.70	82.04
5. Neonatal disorders	0.00	0.00	5. Alzheimer's disease and other dementias	6.62	37.78
6. Stomach cancer	2.33	22.33	6. Liver cancer	2.97	26.98
7. Liver cancer	2.28	20.18	7. Stomach cancer	2.97	30.17
8. Tracheal, bronchus, and lung cancer	26.73	71.05	8. Hypertensive heart disease	0.00	0.00
9. Road injuries	0.18	0.74	9. Road injuries	0.28	1.53
10. Self-harm	0.00	0.00	10. Esophageal cancer	17.87	60.69
11. Hypertensive heart disease	0.00	0.00	11. Colon and rectum cancer	2.67	24.34
12. Drowning	0.00	0.00	12. Lower respiratory infections	20.90	40.25
13. Esophageal cancer	19.45	54.79	13. Chronic kidney disease	0.00	0.00
14. Tuberculosis	4.85	38.74	14. Cirrhosis and other chronic liver diseases	0.00	0.00
15. Alzheimer's disease and other dementias	6.76	37.39	15. Diabetes mellitus	11.45	21.83

*COPD, chronic obstructive pulmonary disease; IHD, ischemic heart disease*.

## Discussion

### Tobacco-Attributable Disease Burdens

Tobacco continued to be the principal cause of death in China. In 2017, ~2.60 million deaths could be attributable to tobacco. And the tobacco-attributable death rates in males increased by 50% from 1990 to 2017. Furthermore, the tobacco-attributable DALY rates were 1489.05 and 6994.02 person-years per 100,000 in females and males, respectively. These figures mean that the health-adjusted life expectancy would increase by ~0.15 and 0.7 years in Chinese females and males, respectively, if tobacco could be controlled effectively. Therefore, China has an enormous tobacco-attributable health burden, and comprehensive and urgent measures are needed to reduce the tobacco-attributable disease burdens in the country.

The sex gap in the tobacco-attributable disease burden was driven by the sex gap in smoking prevalence. The SHS- and chewing tobacco-attributable death rates and DALY rates were close in both sexes, whereas the smoking-attributable death rates and DALY rates in males were considerably higher than in females. Therefore, if smoking prevalence in males could be effectively controlled, then the currently significantly higher tobacco-attributable burden in males may decline to close to that of females. One notable point was that the focus of tobacco control for females changed from SHS in 1990 to smoking and SHS in 2017. In 1990, the SHS-attributable deaths and DALYs accounted for a large proportion of that of tobacco. At this time, the focus of tobacco control on females was SHS. In 2017, the SHS- and smoking-attributable deaths and DALYs were close in females; therefore, we must pay equal attention to smoking and SHS in tobacco control programs.

Moreover, in 2017, the tobacco-attributable deaths in China were concentrated on COPD, IHD, lung cancer, and stroke. From 1990 to 2017, tobacco-attributable deaths occupied a large proportion of the leading causes of death in China, especially in males. For cardiovascular diseases (e.g., stroke and IHD), tobacco-attributable deaths accounted for ~10 and 30% of deaths in females and males, respectively. For lung diseases (e.g., COPD and lung cancer), tobacco-attributable deaths accounted for ~30 and 70% of deaths in females and males, respectively. Tobacco control remains the priority of public health professionals in preventing cardiovascular and lung diseases. Through effective control of tobacco consumption and SHS exposure, some leading causes of death, such as lung cancer and COPD, will become relatively less common in future generations.

### Smoking-Attributable Disease Burdens

In 2017, smoking was the second leading contributor to risk-attributable deaths, and the first for risk-attributable DALYs in China (16). From 1990 to 2017, death and the DALY rates attributed to smoking increased in both sexes. Therefore, a thorough exploration on the smoking-attributable disease burdens in China is urgently needed. Rapid declines in the smoking-attributable disease burdens can be achieved by further reducing the smoking prevalence in China. We obtained the data for the smoking prevalence in China from 1984 to 2018 from the National Prevalence Survey and GATS (6, 17–21). For males, the smoking prevalence was 61.01% in 1984, which peaked in 1996, with 63.00%, and then continuously decreased to 57.4, 52.9, and 50.5% in 2002, 2010, and 2018, respectively. For females, the smoking prevalence was 7.04% in 1984 and then continuously decreased to 3.80 and 2.60% in 1996 and 2002, respectively. After 2002, smoking prevalence in females varied within a narrow range. The smoking prevalence in females was 2.4% in 2010, which decreased to 2.1% in 2018. A significant drop in smoking prevalence was observed from 1990 to 2017 in both sexes. Although under the influences of the demographic aging in China and the long lagged effect of smoking behavior and deaths, the smoking-attributable death rates and DALY rates showed an upward trend in both sexes from 1990 to 2017. Given the lagged effect of smoking behavior and deaths, the smoking-attributable death rates and DALY rates would decline in the future.

To decrease smoking prevalence, it takes multiple efforts: improve smoking-related health education, anti-cigarette trend led by the Chinese government, barrier-free access to smoking cessation counseling, and regulations and restrictions on tobacco advertising, promotion, and sponsorship (TAPS). The most common reason for quitting smoking has always been suffering from smoking-related diseases (22, 23). In 1996, 47.3% of smokers quit due to suffering from smoking-related diseases, and this figure increased to 55.0% in 2010 (22, 24). However, in the future, under the influence of an effective promotion of smoking-related health education, suffering from smoking-related diseases may be replaced by disease prevention as the reason to quit smoking. In 1996, ~40 and 4% of people recognized that lung cancer and heart diseases are related to smoking, respectively (17); in 2017, these figures increased to 82.8 and 50.8%, respectively (6). An improved smoking-related health education makes people more willing to quit smoking, and the anti-cigarette trend led by the Chinese government provided a suitable environment for quitting smoking. Cigarettes play an important role in social contact in the Chinese traditional mindset (25). A Chinese man shares cigarettes to show politeness in social contact; such an act helps them become familiar with one another quickly (26). Moreover, expensive cigarettes are regarded as decent gifts for relatives, friends, or guests (27). Under the influence of such a mindset, quitting smoking is rather difficult because it may affect people's social interaction. However, the anti-cigarette trend led by the Chinese government has been gradually reversing such mindset. In December 2013, the General Offices of the Central Committee of the Communist Party of China and the General Office of the State Council issued a circular message titled “Notice on Leading Cadres Taking the Lead in Smoking Bans in Public Places” (28). The circular message stated that public officials are prohibited from publicly smoking or using public funds to purchase cigarettes, and they should set a positive example in promoting a smoke-free environment and complying with smoking controls. This notice could be seen as a sign of the anti-tobacco trend led by the Chinese government. Under the influence of such anti-tobacco trends, the Chinese traditional mindset toward cigarettes is gradually changing. Quitting smoking was also an important part of reducing the smoking-attributable disease burdens. In 2018, smoking cessation hotlines have been provided to every province in China, and more than 800 smoking cessation clinics have been established (6, 23), which provided great convenience for quitting smoking. As a result, the smoking cessation rates increased significantly from 1984 to 2018. The earliest survey on the rates of smoking cessation in China was in 1984, which showed that the smoking cessation rates were only 4.17% in males and 9.73% in females (18). In surveys following this year, the smoking cessation rates were no longer gender-specific; the total smoking cessation rate was 9.4% in 1996 (17) and increased to 20.1% in 2018 (6). The rising smoking cessation rate in China has effectively reduced the number of people suffering from smoking-attributable disease burdens. Gradual restrictions on TAPS have also contributed remarkably to the decrease in smoking prevalence. TAPS is an important part of tobacco marketing that can add many potential cigarette consumers. The Chinese government enacted the Chinese Advertising Law to restrict TAPS in 1994 (23). In 2015, the revised Chinese Advertising Law prohibited the publication of tobacco advertising in the media, public places, and public transportation (29). In 2016, the China Charity Law stipulated that no organization or individual can use charitable donations to promote tobacco products (6). Most TAPS have been banned after 2016 due to these aforementioned laws.

Smoking prevalence showed a downward trend in both sexes from 1990 to 2018. However, this trend may not continue in the future. A notable fact was that an increasing number of Chinese adolescents have been starting to smoke, especially females. The smoking prevalence in Chinese adolescent females increased from 0.29% in 1984 to 18.1% in 2018; for Chinese adolescent males, smoking prevalence was 26.62% in 1984, which declined to a nadir of 10.86% in 2000, and then increased to 22.3% in 2018 (6, 30). Given the continuously increasing smoking prevalence among Chinese adolescents in the last two decades, the current downward trend of smoking prevalence in adults may reverse as these adolescents grow up. A number of factors have contributed to the increasing trends of smoking prevalence among Chinese adolescents over the past two decades. These factors include inductive tobacco advertisement and promotion, higher life stress accompanied by a fast life pace, and affordable and easily accessible cigarettes. Before 2016, cigarette advertisements and promotion had been numerous in China. These advertisements and promotions linked smoking behavior to being “cool,” “fashionable,” and “elegant,” which may persuade minors to try smoking (31, 32). Besides, some tobacco products claim to help regulate mood, relieve stress, and control weight, which promotes tobacco use in the youth (31). With the high life pressure brought by a fast-paced life, many adolescents may choose to smoke to relax. In addition, cigarettes are easily accessible to adolescents in China. Although the Law of the People's Republic of China on the Protection of Minors prohibits the sale of tobacco products to minors, it is not strictly enforced (33), which makes tobacco products easily accessible to Chinese adolescents. In addition, the influence of the low price of Chinese cigarettes on the smoking prevalence among Chinese adolescents cannot be ignored. In 2000, the cost of 100 packets of cigarettes accounted for 14% of the mean annual income per person in China; nevertheless, this figure decreased to 3% in 2010 (23). In 2018, 1.5% of the gross domestic product per capita was needed to buy 100 packets of cigarettes in China (6). Thus, cigarettes became increasingly affordable for Chinese adolescents from 2000 to 2018. If we hope that the downward trend of smoking prevalence in China will continue in the future, these aforementioned factors should be given attention. In comparison to the smoking prevalence worldwide among males (34.63% in 2016) and females (6.45% in 2016) by the World Bank (34), the smoking prevalence among Chinese females was slightly lower than those worldwide, whereas that of Chinese males was considerably higher. Thus, Chinese tobacco control should still be considerably improved.

The next step in the Chinese tobacco control program should focus on the following aspects: banning cigarette sale to minors, cigarette packaging, and tobacco taxes and prices. As mentioned previously, we should strengthen supervision in the process of cigarette sales and protect minors from cigarettes. Implementing large, graphical health warnings on tobacco products was a crucial part of achieving the WHO Framework Convention on Tobacco Control (WHO FCTC). However, in China, health warnings cover only 30% of the bottom of tobacco packaging, which is well below the 50% recommended by the WHO FCTC (15, 35). These insufficient number of warnings are facing the risk of being canceled in the future (36). Improvement of the proper packaging of cigarettes will help the Chinese government's tobacco control program. In addition to cigarette packaging, tobacco taxes and prices also influence the smoking-attributable disease burdens. Several studies have shown that raising the tobacco taxes and prices may be the most effective policy to reduce tobacco consumption (37–40). On average, a 10% increase in the price of a packet of cigarettes is estimated to reduce ~4% of the demand for cigarettes in high-income countries and ~4–8% in low- and middle-income countries (41). In 2015, the “Notice on adjusting cigarette excise tax” was issued by the Ministry of Finance and the State Administration of Taxation increased the cigarette tax in the process of wholesale from 5% to 11% (42). Nevertheless, the tobacco taxes in China remained lower than the WHO FCTC-recommended benchmark (43). The low tobacco tax led to cigarette prices in China being the lowest worldwide (44), with one Chinese cigarette costing only ~9.9 Chinese Yuan (6). Increasing the tobacco taxes and prices will effectively reduce smoking prevalence, especially among minors. In view of the rapidly increasing smoking prevalence among Chinese minors over the past two decade, increased tobacco taxes and prices may be the most effective measures to achieve the tobacco control goals of “Health China 2030” (45).

In implementing the above measures, especially for tobacco taxes and prices, we must take the effects of tobacco taxes on government revenue into consideration. The China National Tobacco Corporation (CNTC) is a 100% state-owned enterprise that manages and controls the sales of tobacco products in China. The State Tobacco Monopoly Administration (STMA) is a government institution that regulates and supervises the tobacco supply in China. It should be noted that the CNTC and STMA are effectively the same organization, which represents the close connection between the Chinese government and the tobacco industry. The tobacco industry in China is a giant state-owned enterprise, with annual net profits larger than those of the Bank of China or the Petro China Company (23). Hence, increasing tobacco taxes and reducing the profits of the tobacco industry are challenging. To reduce the tobacco tax successfully, the Chinese government needs to exert efforts in two aspects. Firstly, the Chinese government should set up a new tobacco regulation and supervise agencies to separate the sales and monitoring of tobacco. Secondly, the Chinese government should adopt subsidies, production substitution policies, and trade policies to organize and help tobacco growers to switch to alternative crops. Some studies have suggested that if the excise tax per packet of cigarettes increases by 1 Chinese Yuan, then the Chinese government revenues will increase by 65 billion Chinese Yuan (US $9.2 billion), which can be used for health or other government priorities and bring ~9.9 billion Chinese Yuan (US $1.3 billion) of productivity growth to the Chinese economy (46). For long-term economic and healthy development, increased tobacco taxes and prices is imperative.

### SHS-Attributable Disease Burdens

In 2017, SHS was the 13th leading contributor to risk-attributable deaths and the 12th leading contributor to the risk-attributable DALYs in China (16). Contrary to smoking and chewing tobacco, more deaths and DALYs could be attributable to SHS in females than in males in 1990–2017. The inequality of the SHS-attributable disease burdens in both sexes was driven by the difference in smoking prevalence. Smoking prevalence was considerably higher in males (50.5% in 2018) than in females (2.1% in 2018); thus, female non-smokers outnumbered male non-smokers in China. As a result, more female non-smokers were exposed to SHS, which led to more SHS-attributable deaths and DALYs in females. Given the high proportion of SHS-attributable deaths and DALYs to that of tobacco in females, prevention and control of SHS exposure was particularly beneficial to reduce tobacco-attributable disease burdens in females.

The SHS-attributable disease burdens in China declined significantly between 1990 and 2017. From 1990 to 2017, the SHS-attributable DALY rates decreased by ~40%, and the SHS-attributable death rates decreased by about 30% in females and 10% in males. In comparison to the increased trends of smoking-attributable disease burdens, the decreased trends of SHS-attributable disease burdens are a sign of the effective restrictions of SHS exposure in China. Over the past two decades, the decline of SHS exposure in households and public places has played an important role in the decline of the SHS-attributable disease burdens. The total SHS exposure rate was 39.75% in 1984, which increased to 53.5% in 1996 (18). The research by Yang et al. provided the earliest SHS exposure rate by place in China: in 1996, the SHS exposure rates were 71, 32, and 25% in households, public places, and work places, respectively (17). In 2002, the total SHS exposure rate decreased slightly compared with that in 1996, at 51.9% (19). After 2010, we obtained detailed data about SHS exposure. That is, the total SHS exposure rate was 72.4% (47). Thus, ~740 million non-smokers were exposed to SHS in China in 2010, including 64.3% of SHS exposure in households, 60.6% in the workplace, 54.9% in government buildings, 36.8% in health care facilities, 87.6% in restaurants, 34.6% in primary and secondary schools, and 29.0% in public transportations (48). These figures were higher in China than the World Bank's low and lower middle-income countries (49). By 2018, the SHS exposure rates in China showed a comprehensive decline compared with those in 2010, but remained at a high level; these rates included 44.9% of SHS exposure in households, 50.9% in workplaces, 31.1% in government buildings, 24.4% in health care facilities, 73.3% in restaurants, 23.4% in primary and secondary schools, and 12.9% in public transportations (50). GATS2018 also provided SHS exposure rates by gender in households and workplaces; that is, 60.5% for males and 39.6% for females in workplaces and 51.7% for males and 37.9% for females in households. The sharp decline in SHS exposure in households, government buildings, and public transportations was surprising.

Several studies have suggested that the SHS exposure in Chinese females was mainly from households, whereas that in males was mainly from workplaces and public places (17, 19). For females, the SHS exposure rates in households decreased sharply from 71% in 1996 to 37.9% in 2018, which contributed considerably to the decreased SHS-attributable disease burdens in females. A large room for the SHS exposure rate in households to decline cannot be ignored. For males, the decreased SHS-attributable death rates and DALY rates were mainly caused by the decreased SHS exposure rates in public places, such as government buildings, health care facilities, and public transportations. However, the SHS exposure rates were still extremely high in some public places, such as restaurants (73.3% in 2018). In addition to SHS exposure in public places, the high SHS exposure rate of males in workplaces (60.5% in 2018) was associated with the heavy SHS-attributable disease burdens in males. Reducing SHS exposure in households, workplaces, and public places should be the next step in SHS prevention programs.

The Chinese government has been paying increasing attention to the regulation of SHS exposure. In November 2014, the Legislation Office of the State Council sought advices and comments from experts and the public on the draft of the first smoking ban on all indoor and outdoor public places (23). The Central Tobacco Control Program Office used 200,000 public service posters to implement SHS-related health education, reminding everyone of the dangers of SHS (23). Since Beijing began smoking bans in part of public places in 2008, an increasing number of Chinese cities (e.g., Shanghai, Shenzhen, and Harbin) have followed the pace of Beijing and started smoking bans in part of public places (51–53). To date, more than 20 cities have adopted new comprehensive smoke-free policies, which provide protection to about 10% of the Chinese population (6). In November 2014, Beijing passed the strictest smoking control law: smoking bans in all indoor public and working places (23). All of these endeavors suggest that a nationwide comprehensive smoking ban policy is forthcoming.

### Chewing Tobacco-Attributable Disease Burdens

In 2017, chewing tobacco was the 34th leading contributor to risk-attributable deaths and the 43rd leading contributor to the risk-attributable DALYs in China (16). In comparison to smoking and SHS, relatively fewer deaths and DALYs could be attributed to chewing tobacco. Nevertheless, people should still remain cautious about chewing tobacco-attributable disease burdens in the future. E-cigarettes are the most common smokeless tobaccos. Thus far, research on e-cigarettes in China remains limited, even though 95% of the e-cigarettes worldwide are produced in China (54). With cigarettes being increasingly regulated year after year, e-cigarettes have become a possible substitute for cigarettes in China (55). E-cigarettes have become increasingly popular in recent years, especially among the young population, due to their exaggerated marketing (54, 55). The marketing of e-cigarettes claims that they have health-related benefits and no SHS exposure, as well as utility for smoking cessation (56, 57). Furthermore, e-cigarette manufacturers provide them with various tastes and celebrity endorsements, as well as advertisements that link e-cigarettes to fashion to attract potential young consumers (57). The restriction on e-cigarette marketing and selling may be a feasible measure to reduce chewing tobacco-attributable disease burdens. In November 2019, the Chinese government banned the sale of e-cigarettes on the Internet (58, 59), which is a good start to reducing chewing tobacco-attributable disease burdens. In the future, attention should be paid to chewing tobacco in China, in addition to smoking and SHS.

### Limitation

This study still has several limitations. Firstly, this study was based on the GBD 2017 database. Thus, all limitations of GBD 2017 outlined elsewhere are also applicable to the present work. Although the GBD 2017 has made numerous adjustments and corrections to the source, collation, and evaluation of the tobacco-attributable disease burden data to enhance data accuracy and comparability, avoiding inaccuracy thoroughly remains difficult. Secondly, the study only focused on the tobacco-attributable disease burdens from 1990 to 2017 and lacked the data for smoking and chewing tobacco prevalence and SHS exposure. These limitations needed further study to improve.

## Conclusion

Although the tobacco-attributable disease burdens declined rapidly from 1990 to 2017, they remained heavy. Given the high proportion of smoking-attributable deaths and DALYs to that of tobacco, increasing the tobacco taxes and prices may be the most effective and feasible measure to reduce the tobacco-attributable disease burdens. Furthermore, we need to pay attention to e-cigarette marketing and SHS exposure in households, workplaces, and restaurants.

## Data Availability Statement

Publicly available datasets were analyzed in this study. This data can be found here: http://ghdx.healthdata.org/gbd-results-tool.

## Author Contributions

HW and CY contributed to the conception and design of the study. HW organized the database and wrote the first draft of the manuscript. HW and CX performed the statistical analysis. HW, CY, CX, FW, and YW wrote sections of the manuscript. All authors contributed to manuscript revision and read and approved the submitted version.

## Conflict of Interest

The authors declare that the research was conducted in the absence of any commercial or financial relationships that could be construed as a potential conflict of interest.
