# Supplementary material for: Trends in Disease Burden Attributable to Tobacco in China, 1990–2017: Findings From the Global Burden of Disease Study 2017
Source: Front Public Health. 2020 Jul 9;8:237. doi: 10.3389/fpubh.2020.00237 (PMC7381278; doi:10.3389/fpubh.2020.00237)
Supplement: Supplementary file 1 [file Table_1.DOCX]

Supplementary Material

**Supplementary Table 1** The proportion of tobacco-attributable DALYs to total DALYs for top 10 DALY causes in 1990 and 2017

| The proportion of tobacco attributable DALYs to total DALYs in 1990 | | |  | The proportion of tobacco attributable DALYs to total DALYs in 2017 | | |
| --- | --- | --- | --- | --- | --- | --- |
| Top 10 DALYs causes in 1990 | Female | Male |  | Top 10 DALYs causes in 2017 | Female | Male |
| 1 Lower respiratory infections | 22.75% | 23.72% |  | 1 Stroke | 10.66% | 36.67% |
| 2 Neonatal disorders | 0.00% | 0.00% |  | 2 IHD | 16.35% | 45.05% |
| 3 Stroke | 12.88% | 35.68% |  | 3 COPD | 25.95% | 68.22% |
| 4 COPD | 26.48% | 59.21% |  | 4 Tracheal, bronchus, and lung cancer | 28.25% | 80.50% |
| 5 Congenital birth defects | 0.00% | 0.00% |  | 5 Road injuries | 0.20% | 1.20% |
| 6 Road injuries | 0.10% | 0.50% |  | 6 Neonatal disorders | 0.00% | 0.00% |
| 7 IHD | 20.12% | 45.73% |  | 7 Liver cancer | 2.28% | 25.34% |
| 8 Drowning | 0.00% | 0.00% |  | 8 Diabetes mellitus | 15.35% | 27.39% |
| 9 Self-harm | 0.00% | 0.00% |  | 9 Neck pain | 0.00% | 0.00% |
| 10 Diarrheal diseases | 0.00% | 0.00% |  | 10 Depressive disorders | 0.00% | 0.00% |
| 11 Liver cancer | 1.61% | 17.98% |  | 11 Age-related and other hearing loss | 0.00% | 0.00% |
| 12 Stomach cancer | 1.67% | 20.91% |  | 12 Stomach cancer | 2.22% | 29.30% |
| 13 Tuberculosis | 2.88% | 31.79% |  | 13 Low back pain | 3.32% | 33.62% |
| 14 Tracheal, bronchus, and lung cancer | 23.90% | 67.04% |  | 14 Alzheimer's disease and other dementias | 6.47% | 41.34% |
| 15 Depressive disorders | 0.00% | 0.00% |  | 15 Other musculoskeletal disorders | 0.00% | 0.00% |

Note: COPD= Chronic Obstructive Pulmonary Disease, IHD= Ischemic Heart Disease. DALY =disability-adjusted life year.

**Supplementary Figure Legend**

Supplementary Figure 1(a) Top 15 causes of tobacco-attributable DALYs in Chinese female in 1990 and 2017. DALY =disability-adjusted life year.

Supplementary Figure 1(b) Top 15 causes of tobacco-attributable DALYs in Chinese male in 1990 and 2017. DALY =disability-adjusted life year.
