# Supplementary figures and images for: Trends in Disease Burden Attributable to Tobacco in China, 1990–2017: Findings From the Global Burden of Disease Study 2017
Source: Front Public Health. 2020 Jul 9;8:237. doi: 10.3389/fpubh.2020.00237 (PMC7381278; doi:10.3389/fpubh.2020.00237)

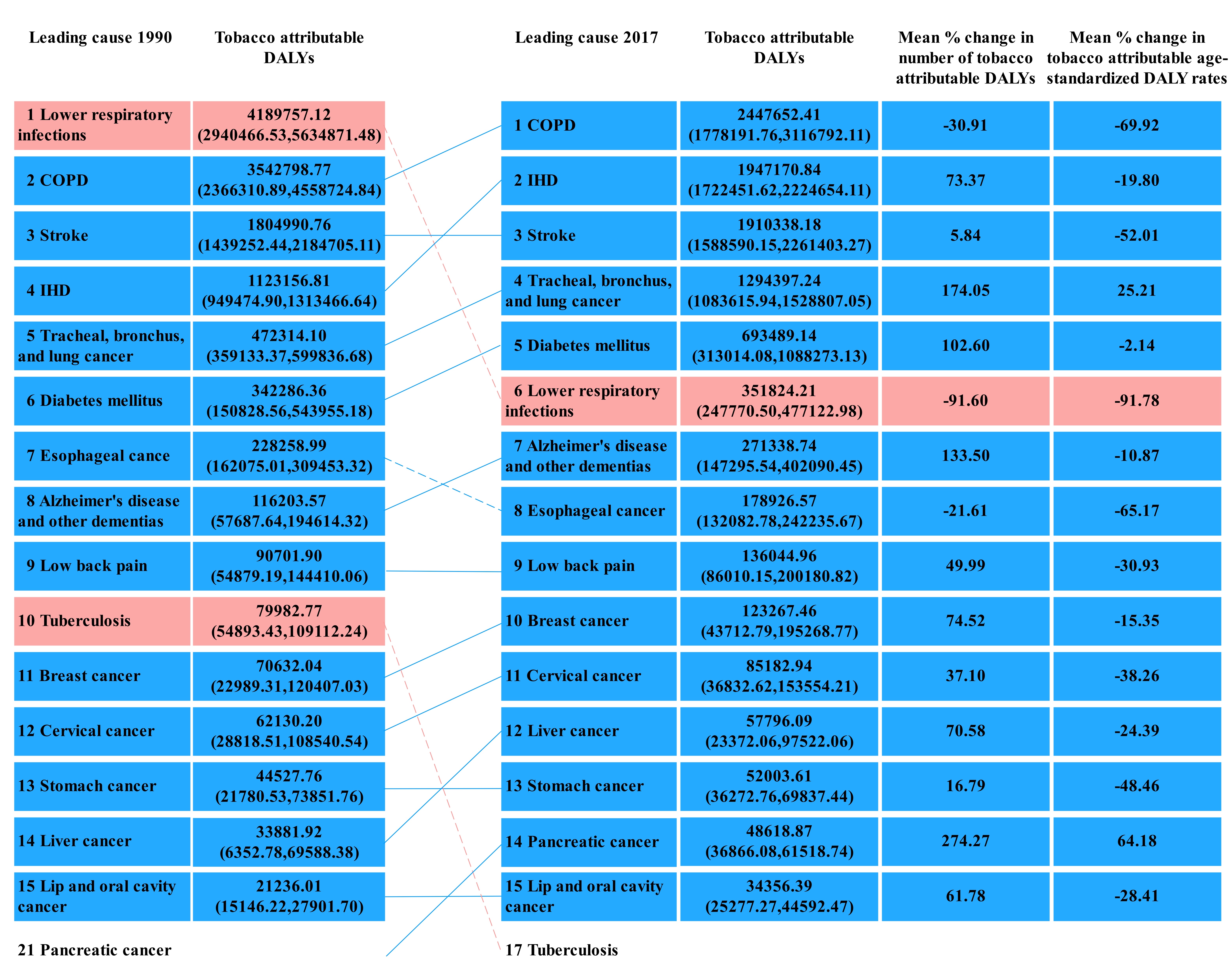

Supplement: Supplementary file 2 [file Image_1.JPEG]

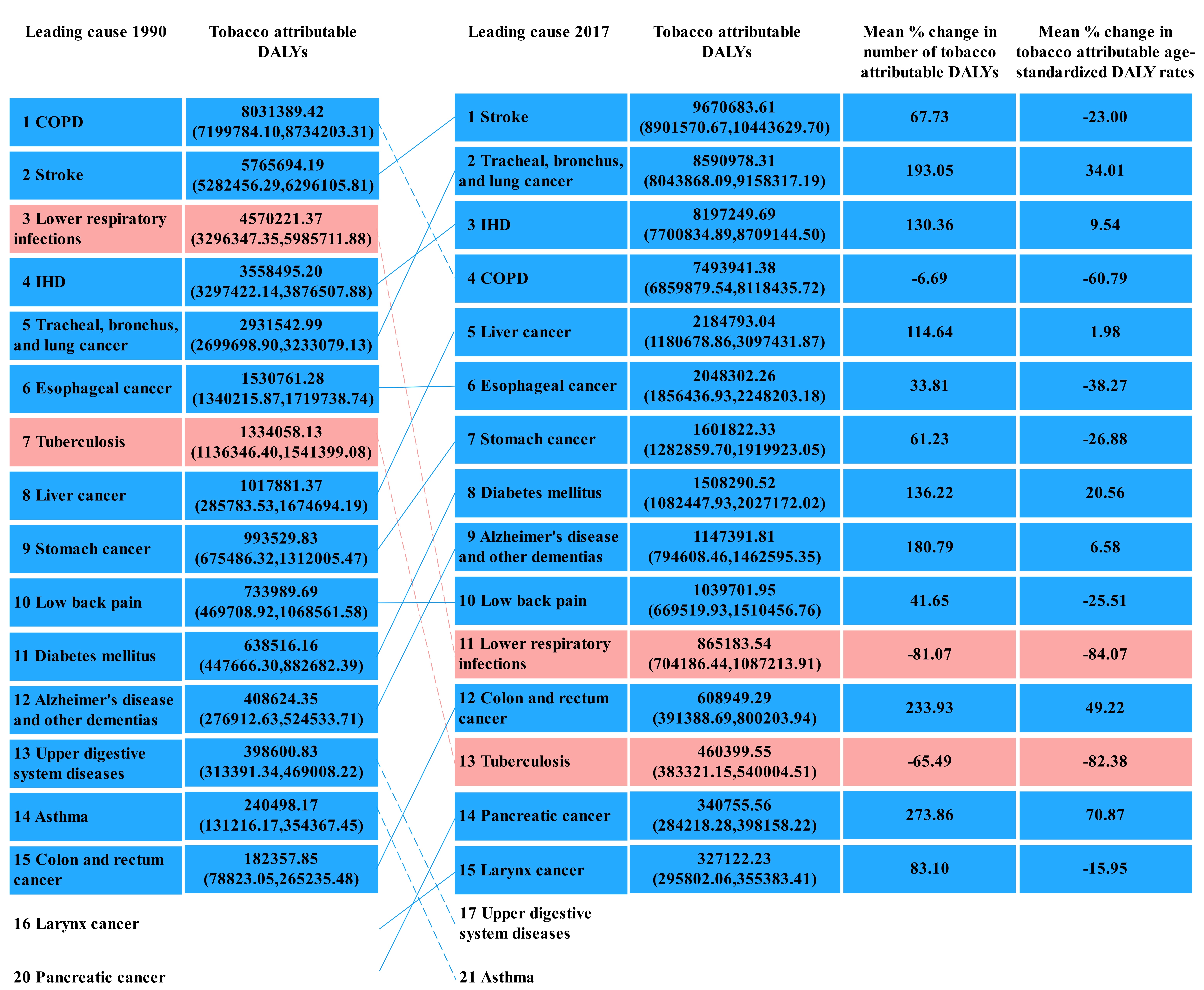

Supplement: Supplementary file 3 [file Image_2.JPEG]
